# A short treatment of cells with the lanthanide ions La^3+^, Ce^3+^, Pr^3+^ or Nd^3+^ changes the cellular chemistry into a state in which RNA replication of flaviviruses is specifically blocked without interference with host-cell multiplication

**DOI:** 10.1099/vir.0.83146-0

**Published:** 2007-11

**Authors:** Gerd Wengler, Gisela Wengler, Andreas Koschinski

**Affiliations:** 1 Institut für Virologie, Fachbereich Veterinärmedizin, Justus-Liebig-Universität, D-35392 Giessen, Germany; 2 Rudolf-Buchheim-Institut für Pharmakologie, Justus-Liebig-Universität, D-35392 Giessen, Germany

## Abstract

Alpha- and flaviviruses contain class II fusion proteins, which form ion-permeable pores in the target membrane during virus entry. The pores generated during entry of the alphavirus Semliki Forest virus have been shown previously to be blocked by lanthanide ions. Here, analyses of the influence of rare earth ions on the entry of the flaviviruses West Nile virus and Uganda S virus revealed an unexpected effect of lanthanide ions. The results showed that a 30 s treatment of cells with an appropriate lanthanide ion changed the cellular chemistry into a state in which the cells no longer supported the multiplication of flaviviruses. This change occurred in cells treated before, during or after infection, did not inhibit multiplication of Semliki Forest virus and did not interfere with host-cell multiplication. The change was generated in vertebrate and insect cells, and was elicited in the presence of actinomycin D. In vertebrate cells, the change was elicited specifically by La^3+^, Ce^3+^, Pr^3+^ and Nd^3+^. In insect cells, additional lanthanide ions had this activity. Further analyses showed that lanthanide ion treatment blocked the ability of the host cell to support the replication of flavivirus RNA. These results open two areas of research: the study of molecular alterations induced by lanthanide ion treatment in uninfected cells and the analysis of the resulting modifications of the flavivirus RNA replicase complex. The findings possibly open the way for the development of a general chemotherapy against flavivirus diseases such as Dengue fever, Japanese encephalitis, West Nile fever and yellow fever.

## INTRODUCTION

Entry of enveloped viruses involves fusion of the envelope with a target membrane. This reaction is regulated by viral fusion proteins. The fusion proteins of alphaviruses (family *Togaviridae*; reviewed by Schlesinger & Schlesinger, 2001) and flaviviruses (family *Flaviviridae*; reviewed by Lindenbach & Rice, 2001) are class II fusion proteins. These proteins have two additional functions: they generate an icosahedral lattice on the viral surface (Lescar *et al.*, 2001; Pletnev *et al.*, 2001) and they form ion-permeable pores in the target membrane during virus entry (Carrasco, 1995; Nyfeler *et al.*, 2001; Wengler *et al.*, 2003). Alphaviruses can be adsorbed to cells, and exposure to low pH activates virus entry at the plasma membrane (White *et al.*, 1980; Paredes *et al.*, 2004). We have previously shown that lanthanide ions block the ion pores generated during entry of alphaviruses at the plasma membrane without interfering with productive infection (Koschinski *et al.*, 2005). As similar ion-permeable pores are generated during entry of the flavivirus West Nile virus (WNV) (Koschinski *et al.*, 2003), we analysed the effect of lanthanide ions on the establishment of a productive infection by flaviviruses. The results of these studies are reported in this paper.

## METHODS

### Viruses and cell cultures.

BHK-21 cells, Vero cells and C2 cells were grown in Dulbecco's modified Eagle's medium (DMEM) with 5 % fetal bovine serum. C2 cells were derived from a cell line established by Singh (1967) from the mosquito *Aedes albopictus* and were adapted to growth in DMEM (Wengler *et al.*, 1978). Semliki Forest virus (SFV) was grown in BHK-21 cells as described previously (Boege *et al.*, 1980). The flaviviruses WNV and Uganda S virus (UGSV) were grown in BHK-21 cells and C2 cells and quantified by plaque assay, as described previously (Wengler *et al.*, 1978), except that plaques were developed by incubation in medium containing 5 % methylcellulose, followed by fixation with formaldehyde and staining with crystal violet.

### Treatment of cells with rare earth ions.

Rare earth elements comprise the lanthanides and the elements scandium (Sc) and yttrium (Y). For rare earth ion treatment, monolayer cultures were incubated with buffer E containing a rare earth ion. Buffer E consisted of 140 mM NaCl, 3 mM KCl, 2 mM MgCl_2_, 2 mM CaCl_2_, 10 mM glucose and 10 mM HEPES (pH 7.4, adjusted with NaOH). Rare earth ions were obtained from Sigma or Aldrich as chloride salts with the following exceptions: promethium is an unstable, radioactive element and was not analysed, and ytterbium (Yb) and lutetium (Lu) were used as trifluoromethanesulfonates. Stock solutions containing rare earth salts at 100 mM concentration in H_2_O were stored at −20 °C. The appropriate buffer E was freshly prepared for each experiment from a solution of buffer E without CaCl_2_ to which the rare earth ion was added first, followed by CaCl_2_. No precipitation of rare earth ions occurred under these conditions. In a typical experiment, the growth medium was discarded, the cell layer washed once with buffer E, and buffer E containing the appropriate rare earth ion was added to the culture, floating on ice. After a 30 s incubation, the solution was removed, the layer washed once with buffer E and the cells were incubated as described in the individual experiments.

### *In vivo* analyses of RNA and protein synthesis.

For determination of protein synthesis, 1.5 cm diameter monolayer cultures were labelled for 30 min with 300 μl growth medium containing 50 μCi of a mixture of [^35^S]methionine and [^35^S]cysteine (Pro-mix I-[^35^S]; Amersham). Cells were suspended in electrophoresis sample buffer and proteins were separated by electrophoresis on pre-cast 10–20 % gradient gels (Cambrex). Gels were stained with Coomassie blue and subjected to autoradiography. RNA was labelled in cells treated with actinomycin D (1 μg ml^−1^) prior to labelling. Ten millilitres of labelling medium contained 1 ml complete growth medium, 9 ml DMEM without phosphate, 0.2 ml fetal bovine serum, 1 μg  actinomycin D ml^−1^ and [^32^P]orthophosphate. RNA was extracted with phenol (pH 5.2) at 60 °C (Scherrer, 1969) and fractionated by centrifugation on 5–20 % (w/w) sucrose density gradients in 50 mM NaCl, 5 mM disodium-EDTA (pH 7.4) in an SW60 Beckman rotor. The absorbance profile was determined in a flow-through cuvette and radioactivity was determined by liquid scintillation counting.

## RESULTS

### Effect of lanthanide ions on virus growth and plaque formation

**Fig. 1. f1:**
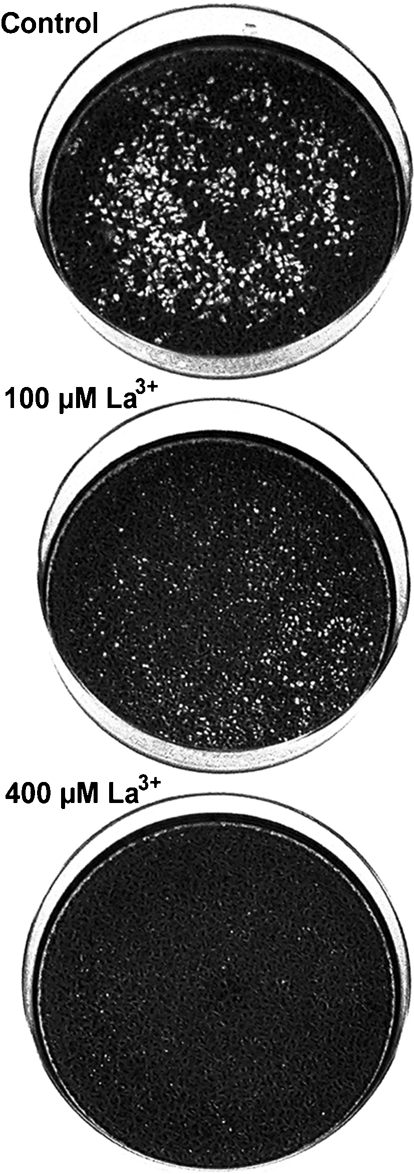
Effect of La^3+^ treatment after virus adsorption on plaque formation. Approximately 200 p.f.u. WNV was adsorbed to BHK cells for 15 min at 0 °C. After adsorption, cells were treated with control solution or with solution containing 50, 100, 200 or 400 μM La^3+^ at 37 °C for 10 s, followed by a 3 day incubation for plaque development. Photographs of the plaques obtained after treatment with control solution, 100 μM La^3+^ or 400 μM La^3+^ are shown.

Experiments were undertaken to analyse a possible inhibitory effect of the presence of lanthanum (La^3+^) ions during entry of WNV. In the experiment shown in Fig. 1 approximately 200 p.f.u. of virus was adsorbed to BHK cell cultures at 0 °C. Solutions containing different concentrations of LaCl_3_ were then added to individual cultures for 10 s and cells were further incubated until plaque formation. It was found that increasing concentrations of La^3+^ led to a reduction in the size of WNV plaques: large plaques were formed in the control culture and small plaques were formed after treatment with 100 μM La^3+^, whilst treatment with 400 μM La^3+^ resulted in only the largest plaques still being detectable as minute plaques. We used a concentration of 500 μM La^3+^ in our standard procedure for La^3+^ treatment, as no plaques were detected after this treatment. In a similar experiment, the size of SFV plaques was not influenced by the addition of La^3+^ ions (data not shown, but see Fig. 3). The reduction in plaque size therefore did not result from a non-specific toxic effect of La^3+^.

**Fig. 2. f2:**
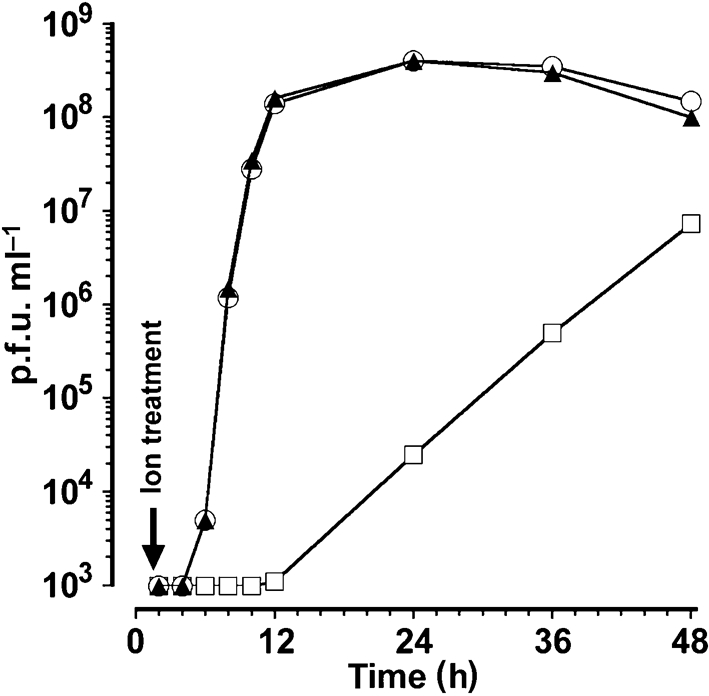
Effect of treatment with La^3+^ or Lu^3+^ after virus entry on the growth of WNV. Three BHK monolayer cultures were infected with WNV at an m.o.i. of approximately 5. At 1.5 h p.i., individual cultures were treated with control solution or with solution containing either 500 μM La^3+^ or 500 μM Lu^3+^ for 10 s at 37 °C and further incubated in growth medium. The growth of infectious virus, as determined by plaque assay, is shown for the control culture (○), La^3+^-treated culture (□) and Lu^3+^-treated culture (▴).

In the above experiments, we were looking for a dose-dependent reduction in plaque number by treatment with La^3+^ during virus entry. The dose-dependent reduction in plaque size was unexpected and was not readily explained by an effect of La^3+^ on virus entry. A possible effect of La^3+^ on virus multiplication at a later step was therefore analysed. The effect of La^3+^ treatment at 1.5 h post-infection (p.i.) on the growth of WNV in BHK cells is shown in Fig. 2. It can be seen that, in the control culture, the exponential phase of virus multiplication was completed by 12 h p.i., whilst no virus production had occurred at this time in cells treated with La^3+^ at 1.5 h p.i. The same results were obtained in UGSV-infected BHK cells (data not shown). These findings showed that La^3+^ treatment interfered with synthesis and/or release of infectious virus particles at a stage later than virus entry or uncoating.

**Fig. 3. f3:**
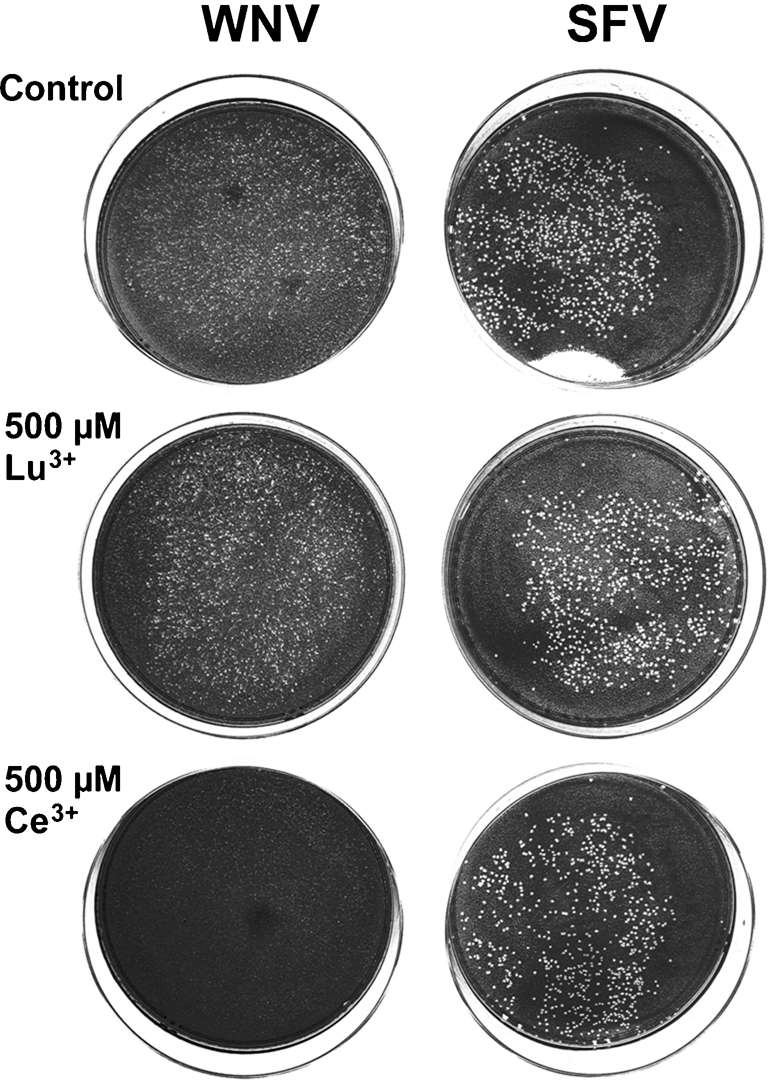
Effect of treatment of cells with lanthanide ions prior to infection on plaque formation by WNV and SFV. The following ions were analysed: La^3+^, Ce^3+^, Gd^3+^, Tb^3+^, Yb^3+^ and Lu^3+^. Fourteen monolayer cultures of Vero cells were used. For each ion, two cultures were treated with 500 μM of the ion for 30 s at 0 °C and further incubated in normal growth medium. As a control, two cultures were treated with solution without a lanthanide ion. After a 4 h incubation in growth medium at 37 °C, one of each culture was infected with approximately 300 p.f.u. WNV or SFV, followed by a 2 day incubation for plaque development. Photographs of plaques generated by WNV after treatment with control solution or with Lu^3+^ or Ce^3+^ are shown on the left. Photographs of the corresponding plaques generated by SFV are shown on the right.

In the experiments reported in Figs 1 and 2, La^3+^ treatment was carried out after virus adsorption. An obvious interpretation of these results is to assume that La^3+^ treatment interferes with the function of a viral protein. In this case, it would be expected that treatment of uninfected cells with La^3+^ prior to infection should not inhibit virus multiplication. Unexpectedly, these experiments showed that treatment of BHK cells with 500 μM La^3+^ 6 h before virus adsorption blocked the development of plaques of WNV and UGSV (data not shown, but see Fig. 3). This type of experiment represents a simple way of determining the effect of variations in experimental conditions on the inhibition of plaque formation. We analysed three questions in such analyses: (i) Is the inhibitory effect specific for BHK cells? (ii) Is the inhibitory effect specific for La^3+^ or can it be obtained with other lanthanide ions? (iii) Is it necessary to perform the La^3+^ treatment at 37 °C or is La^3+^ treatment at 0 °C also effective? Results obtained in an analysis addressing these questions are presented in Fig. 3. In this experiment, Vero cells, which are derived from African green monkeys and which generate plaques when infected with WNV and UGSV, were used instead of BHK-21 cells. The lanthanides comprise the 15 elements between La (atomic no. 57) and Lu (atomic no. 71). Treatment was performed with the lanthanide ions La^3+^, Ce^3+^ (cerium), Gd^3+^ (gadolinium), Tb^3+^ (terbium), Yb^3+^ and Lu^3+^, 4 h before infection by a 30 s incubation at 0 °C. The results showed that treatment with 500 μM La^3+^ or Ce^3+^ blocked the ability of Vero cells to produce WNV plaques. This is shown for Ce^3+^ treatment in Fig. 3. Unexpectedly, it was found that the other four lanthanide ions did not block plaque formation, as shown for Lu^3+^ treatment in Fig. 3. The inactivity of Lu^3+^ ions was further verified in analyses of the effect on single-cycle multiplication of WNV in BHK cells when Lu^3+^ treatment was carried out at 1.5 h p.i. The results showed that Lu^3+^ treatment performed after virus entry also did not inhibit the growth of WNV (Fig. 2). In the experiment shown in Fig. 3, all analyses were performed in parallel with the alphavirus SFV. SFV forms plaques on Vero cells and the results showed that none of the ion treatments inhibited the formation of these plaques. This is shown for the SFV plaques generated in control cultures and after treatment with Ce^3+^ or Lu^3+^ in Fig. 3. Taken together these analyses showed that a 30 s treatment of BHK or Vero cells with La^3+^ or Ce^3+^ at 0 °C, 4 h before virus adsorption, specifically blocked the ability of these cells to produce WNV plaques but did not block the formation of plaques by SFV, and that Gd^3+^, Tb^3+^, Yb^3+^ and Lu^3+^ did not have this activity.

**Fig. 4. f4:**
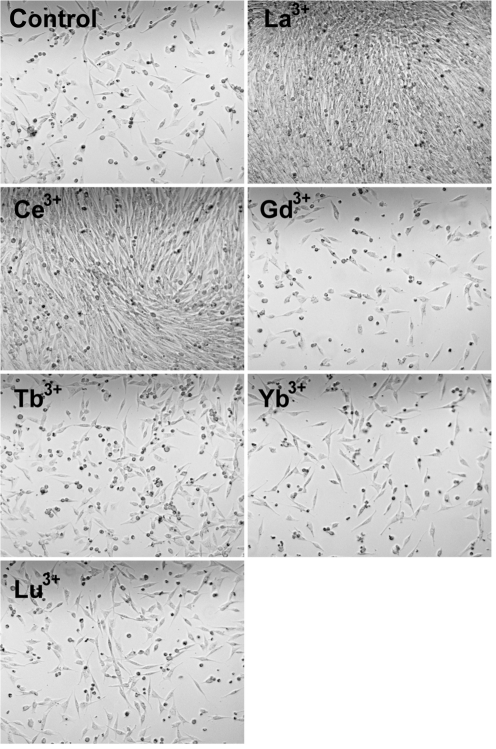
Effect of lanthanide ion treatment on the survival of WNV-infected cells. The ions La^3+^, Ce^3+^, Gd^3+^, Tb^3+^, Yb^3+^ and Lu^3+^ were analysed. Seven freshly confluent BHK cell cultures were infected with WNV at an m.o.i. of approximately 5. At 1.5 h p.i., individual cultures were treated either without lanthanide ion as a control or with a lanthanide ion at 500 μM for 30 s at 0 °C. This treatment was repeated at 12 h time intervals on all cultures. At 30 h p.i., all cultures were trypsinized and reseeded at a 1 : 10 dilution. Photographs (phase contrast, 20× magnification) of all seven cultures taken 18 h after reseeding are shown. The ion analysed is indicated in each photograph.

The data presented in Fig. 2 showed that La^3+^ treatment at 1.5 h p.i. blocked the release of flavivirus particles up to 12 h p.i., but that during the following hours, virus particles began to appear in the growth medium. These results indicated that La^3+^ treatment had a temporary effect on the synthesis of flaviviruses and that a repeated treatment of infected cells every 12 h might induce a continuous state of resistance against flavivirus replication. For plaque assays, virus is adsorbed to subconfluent cell layers, which become confluent during the ensuing 3 days of incubation for plaque development. The finding that lanthanide ion treatment did not inhibit this cell growth, as seen by inspection of the cell layer, indicated that the ion treatment did not generate a cytotoxic effect. Taken together, these data indicated that repeated treatment of infected cell cultures with an appropriate lanthanide ion should specifically interfere with the synthesis of flaviviruses and, at the same time, might allow the survival and multiplication of the infected cells. An experimental analysis of such a system is presented in Fig. 4. BHK cells were infected with WNV and treated at 1.5 h p.i. either with buffer as a control or with a lanthanide ion. This treatment was repeated every 12 h during the following time on all cultures. At 30 h p.i., all cultures were trypsinized and reseeded for further growth at a 1 : 10 dilution. Photographs of all cultures 18 h after reseeding are shown in Fig. 4. It can be seen that few cells survived this procedure in the infected control culture and in the infected cultures treated with Gd^3+^, Tb^3+^, Yb^3+^ or Lu^3+^, whereas the infected cells treated with La^3+^ or Ce^3+^ grew into an apparently intact confluent culture of adherent cells, even after a 1 : 10 dilution. These data led to the important conclusion that the appropriate ion treatment inhibits the multiplication of flaviviruses and does not interfere with host-cell multiplication.

In the experiments reported above, sparse cultures of WNV-infected BHK cells, treated with La^3+^ or Ce^3+^ at 1.5 h p.i., grew into confluent monolayers without any visible cytolytic effect. This system represents a simple assay to analyse the ability of all rare earth ions to inhibit the replication of flaviviruses. The rare earth ions comprise the lanthanide ions and the ions Sc^3+^ and Y^3+^. All rare earth ions with the exception of promethium, which is an unstable, radioactive element, were analysed. It was found that La^3+^ and Ce^3+^ blocked the development of the virus-induced cytolytic effect, Pr^3+^ (praseodymium) and Nd^3+^ (neodymium) were slightly less active, Sm^3+^ (samarium) had at best minimal activity and that all other rare earth ions had no discernible activity (data not shown).

### Effect of lanthanide ions on the synthesis of virus-specific molecules

**Fig. 5. f5:**
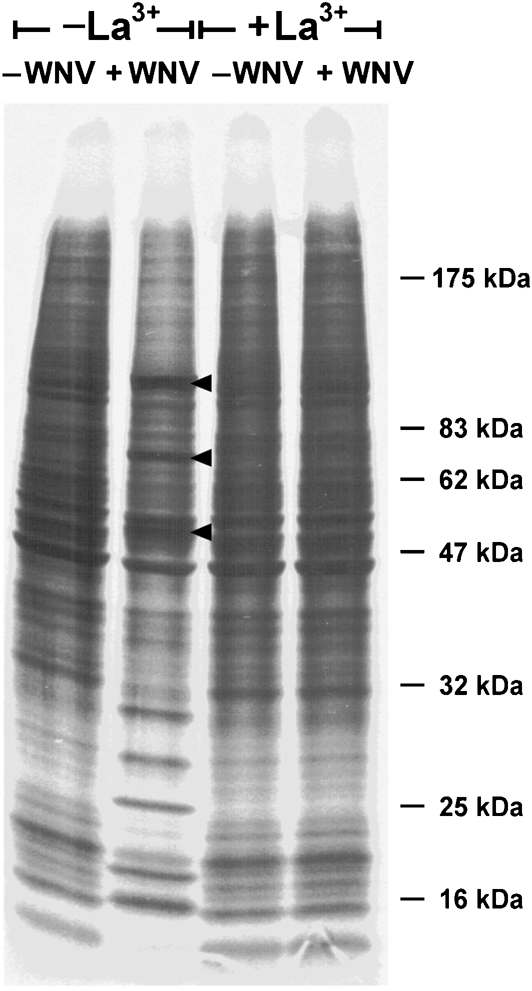
Effect of La^3+^ treatment after virus entry on the synthesis of WNV-specific proteins. Four freshly confluent monolayer cultures of BHK cells were treated as follows: two cultures were infected with WNV at an m.o.i. of approximately 5 and two cultures were mock-infected. At 1.5 h p.i., one of the infected cultures was treated with La^3+^ for 30 s at 0 °C and the other infected culture was treated with control solution without La^3+^. The mock-infected cultures were treated in the same manner. After a further 10 h incubation under conditions for growth, the cells were subjected to a 30 min labelling with ^35^S-labelled amino acids and harvested. Cells were suspended in electrophoresis sample buffer and subjected to SDS-PAGE on a 10–20 % gradient gel. An autoradiogram of this gel is shown. The positions of the virus-encoded proteins NS5 (97 kDa), NS3 (74 kDa) and E (50 kDa) (Castle *et al.*, 1986) are indicated by arrowheads. The migration of marker proteins is also indicated.

The above analyses indicated that ion treatment of cells blocked their ability to produce infectious virus at a step later than entry and uncoating. Flaviviruses accumulate as non-infectious particles in intracellular vacuoles and are activated during release (reviewed by Lindenbach & Rice, 2001). The experiment shown in Fig. 5 analysed whether La^3+^ interfered with one of these later steps. Mock-infected or WNV-infected BHK cells were treated with control solution or with 500 μM La^3+^ at 1.5 h after infection or mock-infection, respectively, and the patterns of proteins synthesized 10 h later were analysed by pulse-labelling with ^35^S-labelled amino acids and SDS-PAGE. It can be seen that, in the absence of La^3+^ treatment in mock-infected cells, a complex pattern of proteins was produced, whereas in infected cells a different pattern containing dominant bands of virus-specific proteins was produced. In contrast, in La^3+^-treated cells, the pattern of proteins synthesized in mock-infected and in infected cells was identical. Furthermore, the pattern of proteins synthesized in untreated mock-infected cells, in La^3+^-treated mock-infected cells and in La^3+^-treated infected cells were identical. Thus, the data showed that treatment of infected cells with La^3+^ at 1.5 h p.i. specifically blocked the synthesis of viral proteins and that this effect did not result from a general toxicity of La^3+^ treatment.

**Fig. 6. f6:**
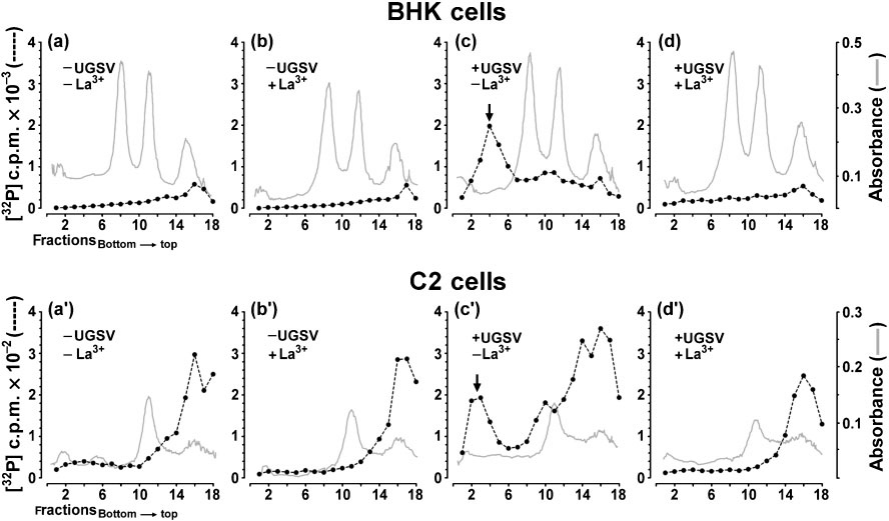
Effect of La^3+^ treatment after virus entry on the synthesis of UGSV-specific RNA in BHK vertebrate and C2 insect cells. For each cell type, four monolayer cultures were treated as follows: two cultures were infected with UGSV at an m.o.i. of approximately 5, two cultures were mock-infected and all cultures were incubated in growth medium at 37 °C. Actinomycin D was added to all cultures to a final concentration of 1 μg ml^−1^ at 1 h p.i. After 30 min, one of the two infected cultures was treated with La^3+^ ions (500 μM, 30 s, 0 °C) and the second infected culture was treated with buffer without La^3+^ as a control. The two mock-infected cultures were treated in the same manner. All cultures were further incubated at 37 °C with growth medium containing actinomycin D. At 10 h p.i., all cultures were subjected to labelling with ^32^P for 0.5 h and harvested. RNA was isolated by phenol extraction and fractionated by centrifugation on sucrose density gradients as described in Methods. The absorbance profile (continuous line) and the distribution of acid-precipitable radioactivity (dotted line) in the gradients are shown. RNA isolated from BHK cells (a–d) and from C2 cells (a′–d′) is shown. The treatment of cultures from which the RNA was derived is indicated in the figure. The absorbance profile represents the sedimentation of the rRNA. The peak containing the 42S UGSV genome RNA is indicated by an arrow.

All flavivirus-specific proteins are synthesized from a 42S RNA, which also serves as the viral genome. The data reported above therefore led to the question of whether ion treatment induced a block in synthesis of 42S RNA and therefore of the synthesis of virus-specific proteins. In principle, the experiment shown in Fig. 5 was repeated but the viral RNA was labelled and the pattern of labelled RNA was analysed. Alpha- and flaviviruses replicate in cells in which the transcription of cellular DNA is blocked by actinomycin D. No virus-specific RNA was synthesized in UGSV-infected BHK cells treated with La^3+^ at 1.5 h p.i., which had been labelled with ^32^P at 10 h p.i. after the addition of actinomycin D at 9.5 h p.i. (data not shown). In this type of experiment, ion treatment was performed prior to the addition of actinomycin D and therefore might possibly influence the transcription of cellular DNA. These considerations led to the question of whether the La^3+^ effect was also elicited if cellular transcription was blocked prior to La^3+^ treatment. Therefore, in the experiment shown in Fig. 6, La^3+^ treatment was performed after the addition of actinomycin D. In this experiment, the influence of La^3+^ treatment at 1.5 h p.i. on the synthesis of UGSV-specific RNA was analysed both in BHK-21 vertebrate cells and in C2 insect cells. The results of analyses of RNA molecules synthesized in BHK cells are shown in Fig. 6(a–d). As expected, in mock-infected, untreated cells (Fig. 6a[Fig f6]) and in mock-infected, La^3+^-treated cells (Fig. 6b[Fig f6]), no sedimentable RNA was synthesized in the presence of actinomycin D. In untreated, infected cells (Fig. 6c[Fig f6]), 42S RNA viral genome and mRNA, and a 20S complex replicative RNA were synthesized (Wengler *et al.*, 1978). The most important result (Fig. 6d[Fig f6]) showed that treatment of infected cells with La^3+^ ions blocked the synthesis of both virus-specific RNA species. As replication of most flaviviruses in insect cells does not generate a cytolytic effect, plaque assay analyses similar to those presented in Figs 1 and 3 are not readily possible in insect cells, but RNA labelling experiments can easily be performed in these cells. The pattern of RNA molecules synthesized in C2 insect cells are shown in Fig. 6(a′–d′). The data showed that La^3+^ treatment also induced a block in viral RNA synthesis in these cells. Taken together, these data showed that La^3+^ treatment induced a block in viral RNA synthesis that was independent from the transcription of cellular genes and that this induction occurred in both vertebrate and insect cells.

**Fig. 7. f7:**
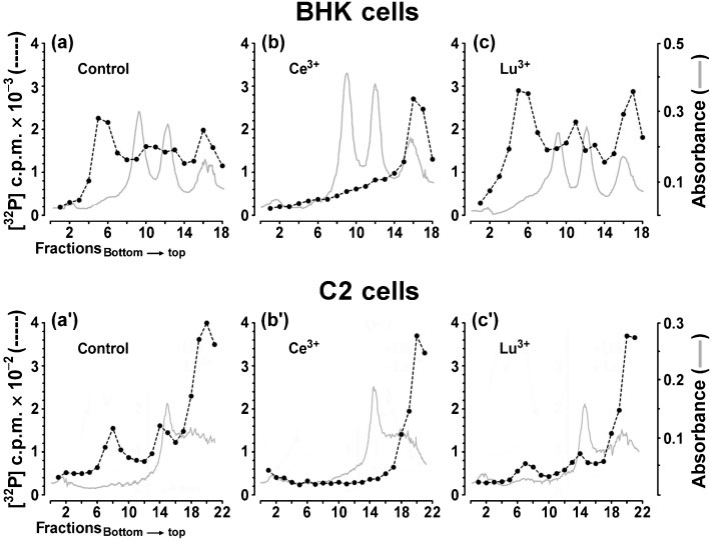
Effect of lanthanide ion treatment after virus entry on the synthesis of UGSV-specific RNA in vertebrate and insect cells. The ions La^3+^, Ce^3+^, Gd^3+^, Tb^3+^, Yb^3+^ and Lu^3+^ were used. The following three virus/cell systems were analysed: UGSV-infected BHK-21 cells, UGSV-infected C2 cells and SFV-infected BHK-21 cells. For each virus/cell system, seven monolayer cultures were infected at an m.o.i. of approximately 5 and treated with actinomycin D (1 μg ml^−1^) at 1 h p.i. At 1.5 h p.i., one culture of each virus/cell system was subjected to treatment with control solution and the six other cultures were treated by a 30 s incubation at 0 °C with a lanthanide ion at 500 μM. At 8.5 h p.i., all 21 cultures were labelled with ^32^P and the RNA was extracted and analysed by centrifugation as described in the legend to Fig. 6. RNA isolated from UGSV-infected BHK cells treated with control solution or with Ce^3+^ or Lu^3+^ is shown in (a), (b) and (c), respectively. RNA derived from UGSV-infected C2 cells treated with control solution or with Ce^3+^ or Lu^3+^ is shown in (a′), (b′) and (c′), respectively.

The analyses of the effects of rare earth ions on flavivirus-infected BHK cells described above showed that treatment with La^3+^, Ce^3+^, Pr^3+^ or Nd^3+^ blocked the replication of flaviviruses effectively, that Sm^3+^ had a small effect and that the other rare earth ions had no discernible effect. During further analyses of the effect of ion treatment on the synthesis of viral RNA, it was observed that the sharp distinction between active and inactive ions observed in BHK cells was not found in C2 insect cells (Fig. 7). UGSV-infected BHK cells, UGSV-infected C2 cells and SFV-infected BHK cells were used in the three parts of this experiment. For each part of the experiment, seven monolayer cultures were infected and treated with actinomycin D at 1 h p.i. At 1.5 h p.i., the seven cultures were treated either with control solution or with La^3+^, Ce^3+^, Gd^3+^, Tb^3+^, Yb^3+^ or Lu^3+^. The RNA synthesized in these cultures between 8.5 and 9 h p.i. was labelled with ^32^P and analysed by centrifugation, resulting in a total of 21 RNA analyses. Six of these analyses are presented in Fig. 7. Data derived from the seven SFV-infected BHK cell samples are not shown, as none of the six ions had an inhibitory effect on the synthesis of SFV-specific RNA. The analyses of RNA derived from UGSV-infected BHK cells showed that, in accordance with the analyses described above, La^3+^ and Ce^3+^ blocked the synthesis of virus-specific RNA, whereas treatment with Gd^3+^, Lu^3+^, Tb^3+^ or Yb^3+^ had no inhibitory effect. The control analysis (Fig. 7a[Fig f7]) and the effect of Ce^3+^ (Fig. 7b[Fig f7]) and Lu^3+^ (Fig. 7c[Fig f7]) are shown. However, in UGSV-infected C2 insect cells, all four ions that were inactive in vertebrate cells had a significant inhibitory activity and reduced the synthesis of UGSV-specific RNA by approximately 50 %. As an example, the control analysis (Fig. 7a[Fig f7]′), the effect of Ce^3+^ (Fig. 7b[Fig f7]′) and the effect of Lu^3+^ (Fig. 7c[Fig f7]′) are shown.

**Fig. 8. f8:**
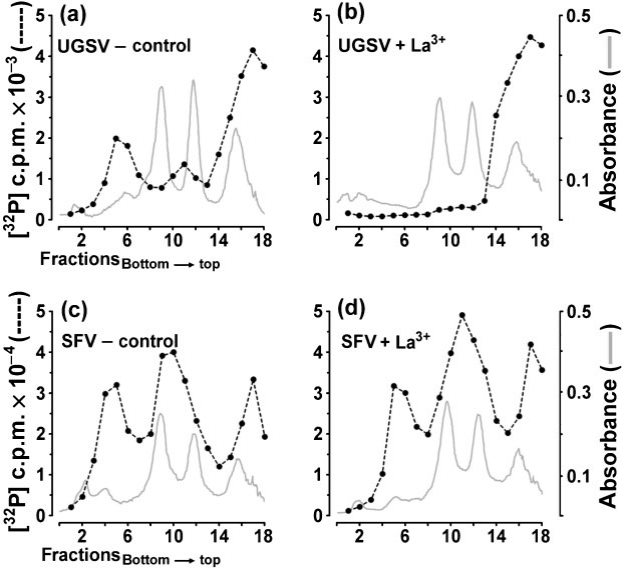
Effect of La^3+^ treatment performed at different times during virus multiplication on viral RNA synthesis. Twelve cultures of freshly confluent BHK cells were treated as follows: six cultures were each infected with UGSV or SFV at an m.o.i. of approximately 5 and incubated in growth medium containing actinomycin D at 37 °C. At 1.5, 3.5, 5.5, 7.5 and 9.5 h p.i., one UGSV-infected and one SFV-infected culture were treated with 500 μM La^3+^ for 30 s at 0 °C. As controls for both virus systems, one infected culture was treated with control solution at 9.5 h p.i. All cultures were labelled with ^32^P at 10.5 h p.i. for 1 h. RNA was extracted and analysed by centrifugation as described in the legend to Fig. 6. The analyses of RNA derived from UGSV-infected cells treated with control solution or with La^3+^ at 9.5 h p.i. are shown in (a) and (b), respectively. The corresponding analyses of RNA isolated from SFV-infected cells are shown in (c) and (d).

In the experiments reported above, treatment with lanthanide ions was performed no later than 1.5 h p.i. At this time, no detectable amount of viral replication complexes has been assembled. Inhibition of viral RNA synthesis therefore could result from a block in the assembly of replication complexes, whereas functional complexes might be unaffected. An experiment to analyse the effect of lanthanide ion treatment on the activity of functional polymerase complexes is shown in Fig. 8. UGSV-infected BHK cells were treated with La^3+^ ions at different times during the exponential phase of virus multiplication. In all cultures, RNA was labelled at the end of the exponential growth phase between 10.5 and 11.5 h p.i. Experiments were performed in parallel in BHK cells infected with SFV. Analyses of RNA molecules synthesized in UGSV-infected cells treated at 9.5 h p.i. with control solution or with La^3+^ are shown in Fig. 8(a, b), respectively. These data showed that no labelled virus-specific RNA could be detected in the La^3+^-treated cells. In contrast, La^3+^ treatment did not inhibit the RNA synthesis of the alphavirus SFV (Fig. 8c, d). These results indicated that lanthanide ion treatment can specifically inactivate functional flavivirus replication complexes.

In the RNA labelling experiments reported above, labelling times that were long compared with the time of synthesis of individual RNA molecules were used. Therefore, the absence of labelled RNA could have resulted from rapid degradation of newly synthesized RNA. The stability of virus-specific RNA in cells treated with lanthanide ions was therefore analysed in an experiment in which virus-specific RNA was labelled first, followed by lanthanide ion treatment and RNA analyses. The finding that the same patterns of labelled RNA were obtained from cultures treated with control solution and from ion-treated cultures (data not shown) showed that treatment of cells containing radioactively labelled RNA with lanthanide ions did not induce degradation of virus-specific RNA.

## DISCUSSION

The flaviviruses UGSV and WNV used in the above experiments belong to two different serocomplexes and we believe that it is reasonable to assume that the results obtained in our experiments are representative of flaviviruses in general. The data described indicate that a short treatment of vertebrate or insect cells with appropriate lanthanide ions induces a change in cellular metabolism that blocks the ability of the cell to support the synthesis of flavivirus RNA. The data presented in Fig. 2 indicated that the block was a temporary effect and the data presented in Fig. 4 showed that repeated treatment of flavivirus-infected cells every 12 h with an active lanthanide ion established a state of resistance to viral replication without interfering with multiplication of the infected host cells, which grew into confluent monolayers even after a 1 : 10 dilution of the cultures. This experiment constitutes clear evidence that even repeated lanthanide ion treatment, performed according to our procedure, had no significant cytotoxic effect on the host cells. The comparative analyses of ^35^S-labelled proteins synthesized in untreated and ion-treated cells (Fig. 5) supported this conclusion using a biochemical technique. In view of this situation, we made no further quantitative analyses of any possible cytotoxic effect generated by our ion treatment procedure.

Lanthanide ions block ion flow through the pores generated in the target membrane during entry of alphaviruses (Koschinski *et al.*, 2005). The effect of lanthanide ions described above had no recognizable relationship to this block, as it could be induced by treating infected cells with lanthanide ions many hours after virus entry.

Treatment of cells with interferon induces a state of resistance expressed after virus entry. The block in virus replication induced by lanthanide ions was different from interferon-induced resistance, as induction of resistance by interferon involves transcription of cellular genes, whereas lanthanide ion treatment was effective in the presence of actinomycin D. Furthermore, virus infection of interferon-treated cells leads to degradation of viral and cellular mRNA and to cell death, whereas infection of lanthanide ion-treated cells left the translation of cellular mRNA unchanged and allowed multiplication of cells after infection.

We do not know how lanthanide ions exert their inhibitory effect, but the data reported above suggest the following: (i) Lanthanide ions react with the surface of the target cell in a reaction that is also effective at 0 °C. The nature of this reaction is not known. It may involve an interaction with a receptor or entry of lanthanide ions through the plasma membrane into the cytoplasm. (ii) This reaction induces a change in the cellular chemistry, which includes the inactivation of a component of the RNA replication complex of flaviviruses. Inactivation is achieved if treatment is performed before virus infection, during virus infection or after virus infection. (iii) This inactivation does not depend on transcription of the host genome. (iv) The process is conserved between vertebrate and insect cells.

In vertebrate cells, La^3+^ and Ce^3+^ blocked the development of the virus-induced cytolytic effect, Pr^3+^ and Nd^3+^ were less active, Sm^3+^ had at best minimal activity and all other rare earth ions had no discernible activity. With increasing atomic number, the diameter of lanthanide ions decreases. The data showed that the activity of the lanthanide ions decreased with decreasing diameter. In vertebrate cells, only the four ions with the largest diameter could effectively induce virus resistance. This strong selectivity was not observed in insect cells, in which even Lu^3+^, the smallest lanthanide ion, had an inhibitory effect.

Flaviviruses cause important diseases such as dengue, yellow fever and various encephalitides (reviewed by Burke & Monath, 2001). Currently, specific chemotherapy is not available against any disease caused by a flavivirus (reviewed by Leyssen *et al.*, 2003). According to the Merck index, the LD_50_ of LaCl_3_ . 7H_2_O in rats after intraperitoneal administration is 350 mg kg^−1^. As LaCl_3_ . 7H_2_O has a molecular mass of 371 g mol^−1^, this LD_50_ corresponds to a concentration of about 1 mM La^3+^ after homogeneous distribution in the animal. These data indicate that, if an appropriate route of administration and an appropriate time schedule are chosen, lanthanide ions might represent effective drugs for the treatment of flavivirus diseases. It is to be expected that the host-cell function targeted by lanthanides is used by all flaviviruses and that therefore appropriate lanthanide ions will have a broad spectrum of activity against flaviviruses.

The experiments reported above will lead to studies in four different new directions: (i) characterization of the biochemical effects of active lanthanide ions on uninfected cells; (ii) analyses of alterations induced by these treatments in the RNA replication complex of flaviviruses; (iii) the search for an inhibitory activity of lanthanide ion treatment on the replication of other viruses; and (iv) analyses of the use of lanthanide ions in a broad-spectrum, host-cell-based chemotherapy against flaviviruses and possibly other viruses.
